# ER stress‐related ATF6 upregulates CIP2A and contributes to poor prognosis of colon cancer

**DOI:** 10.1002/1878-0261.12365

**Published:** 2018-08-20

**Authors:** Chun‐Yu Liu, Chia‐Chi Hsu, Tzu‐Ting Huang, Chia‐Han Lee, Ji‐Lin Chen, Shung‐Haur Yang, Jeng‐Kai Jiang, Wei‐Shone Chen, Kuan‐Der Lee, Hao‐Wei Teng

**Affiliations:** ^1^ Division of Medical Oncology Department of Oncology Center for Immuno‐Oncology Taipei Veterans General Hospital Taiwan; ^2^ School of Medicine National Yang‐Ming University Taipei Taiwan; ^3^ Comprehensive Breast Health Center Taipei Veterans General Hospital Taiwan; ^4^ Division of Transfusion Medicine Department of Medicine Taipei Veterans General Hospital Taiwan; ^5^ Division of Colon and Rectum Surgery Department of Surgery Taipei Veterans General Hospital Taiwan; ^6^ Department of Surgery National Yang‐Ming University Hospital Yilan Taiwan; ^7^ Division of Hematology and Oncology Department of Internal Medicine Taipei Medical University Hospital Taiwan; ^8^ School of Medicine Taipei Medical University Taiwan

**Keywords:** ATF6, CIP2A, colon cancer, ER stress

## Abstract

Endoplasmic reticulum (ER) stress is an adaptive response to various stress conditions and plays emerging roles in cancer. Activating transcription factor 6 (ATF6), one of the three major ER stress transducers, has been shown to contribute to chemoresistance by altering cancer cell survival. Cancerous inhibitor of protein phosphatase 2A (CIP2A) is an oncogene, and its expression has been correlated with the prognosis of patients with cancer. In this study, we aimed to explore the relationship between ER stress‐related ATF signaling and CIP2A. We found that CIP2A expression was positively correlated with ATF6 expression by analyzing publicly available RNA sequence data of patients with colorectal cancer (The Cancer Genome Atlas, TCGA). In addition, we demonstrated that tunicamycin‐induced ER stress *in vitro* upregulated ATF6 and CIP2A. Mechanistically, we found that ATF6 directly bound to the CIP2A promoter and induced CIP2A gene expression, which contributed to colon cancer cell survival. Furthermore, knockdown of CIP2A reduced the viability of cells under ER stress. Most importantly, immunohistochemical analysis of a tissue microarray from a colon cancer patient cohort showed that higher expression levels of ATF6 and CIP2A were associated with a trend toward poor prognosis. Taken together, our results show that ER stress‐related ATF6 upregulates CIP2A and contributes to the prognosis of colon cancer. Targeting CIP2A may disrupt ER stress‐mediated colon cancer cell survival and thus improve the prognosis of patients with colon cancer.

**Abbreviations**
5‐FU: 5‐fluorouracil
ATF6: activating transcription factor 6
CIP2A: cancerous inhibitor of protein phosphatase 2A
CRC: colorectal cancer
ER: endoplasmic reticulum
IHC: immunohistochemistry
IRE1: inositol‐requiring enzyme 1
OS: overall survival
PERK: PKR‐like ER kinase
PVDF: polyvinylidene fluoride
TCGA: The Cancer Genome Atlas

## 1 Introduction

Colorectal cancer (CRC) is the third most common cancer and the third leading cause of cancer death worldwide, with rising incidence among patients younger than 55 (Siegel *et al*., 2017). Despite the success in prolonging the overall survival (OS) via treatment with the multitargeted inhibitor regorafenib (Grothey *et al*., 2013), metastatic CRC remains a challenge, with a median OS in patients with metastatic CRC of approximately 30 months (Falcone *et al*., 2007; Grothey *et al*., 2013). Therefore, there is a need to find new targets and strategies for treating patients with metastatic CRC.

Endoplasmic reticulum (ER) stress is an adaptive response to intracellular stresses, including unfolded protein response (UPR), imbalance of redox state, perturbation in calcium (Ca^2+^) homeostasis, and nutrient deprivation (Hetz, 2012; Ron and Walter, 2007). ER stress signaling is regulated by three major stress transducers located in the ER membrane, activating transcription factor 6 (ATF6), inositol‐requiring enzyme 1 (IRE1), and PKR‐like ER kinase (PERK), and has been implicated in diverse diseases such as cancer, type 2 diabetes mellitus (T2DM), and neurodegenerative and rheumatic diseases (Cnop *et al*., 2012; Navid and Colbert, 2017; Oakes and Papa, 2015). The roles of PERK‐ and IRE1‐mediated ER stresses in the regulation of survival were controversial and were thought to be dependent on cell types and stress conditions, as well as the microenvironment (Clarke *et al*., 2014; Garg *et al*., 2015). In contrast to PERK and IRE1, ATF6 was primarily identified as a cytoprotective role during ER stress (Wu *et al*., 2007; Yamamoto *et al*., 2007). The ER membrane‐anchored ATF6 was activated by proteolysis and acted as transcriptional factor for regulating downstream expression of genes responsible for stresses (Ron and Walter, 2007; Wang *et al*., 2000). Growing evidence in various cancer types has shown that activated ATF6 signaling is correlated with lower OS, cancer recurrence, metastatic lesions, tumor growth, and resistance to radiation therapy and chemotherapy (Dadey *et al*., 2016; Higa *et al*., 2014), indicating that targeting ATF6 signaling might be a strategy for the treatment of cancer. However, the role of ATF6 in colon cancer and the detailed mechanism by which ATF6 regulates colon cancer survival are poorly understood.

Cancerous inhibitor of protein phosphatase 2A (CIP2A, also known as p90 tumor‐associated antigen or KIAA1524) has been reported to be overexpressed in cancer and is an emerging predictor for prognosis in various cancer types including CRC (Bockelman *et al*., 2011; Dong *et al*., 2011; Liu *et al*., 2014; Teng *et al*., 2012). CIP2A exerts its role as oncoprotein by suppressing protein phosphatase 2A (PP2A) and subsequently activating oncogenic proteins, such as Akt, c‐Myc, and mitogen‐activated protein kinase (Chen *et al*., 2010; Mumby, 2007; Tseng *et al*., 2012). Recent studies showed that CIP2A regulates cell proliferation and migration in many types of cancer (Junttila *et al*., 2007). Moreover, our previous studies also revealed that CIP2A plays a critical role in the sensitivity of TD52 (an erlotinib derivative) as well as bortezomib in breast cancer and liver cancer (Chen *et al*., 2010; Liu *et al*., 2017; Tseng *et al*., 2012), suggesting that CIP2A signaling is a promising target for cancer therapy. Moreover, CIP2A is highly associated with the EGFR pathway in CRC (Markowitz and Bertagnolli, 2009). CIP2A was a poor prognostic marker in CRC, and it acts as an oncogene. Regarding drug resistance, we found that CIP2A is associated with drug resistance in CRC, including resistance to 5‐fluorouracil (5‐FU), irinotecan, oxaliplatin, and cetuximab (Teng *et al*., 2012; Wang *et al*., 2014). Therefore, it is imperative to explore the underlying mechanisms by which CIP2A exerts its oncogenic signaling.

In this study, we evaluated the correlation between ATF6 and CIP2A in patients with colon cancer. In addition, we also found that ATF6‐like binding element could be observed within the CIP2A promoter, suggesting that ATF6 might control the expression of CIP2A through transcriptional regulation. Moreover, the correlation between ATF6‐CIP2A signaling and the OS in patients with colon cancer was further investigated.

## 2 Materials and methods

### 2.1 Cell cultures

The human embryonic kidney cell line (HEK293T) and human colon cancer cell lines CaCO_2_, and SW480 (American Type Culture Collection, ATCC, Manassas, VA, USA) were maintained in Dulbecco's modified Eagle medium (Gibco, Grand Island, NY, USA). The human colon cancer cell line, HCT‐15, was maintained in RPMI‐1640 (Gibco). All cell lines were maintained in media supplemented with 10% fetal bovine serum (Hyclone, Logan, UT, USA) in a 37 °C humidified incubator and an atmosphere of 5% CO_2_ in air.

### 2.2 Antibodies and reagents

Antibodies against ATF6, CIP2A, GRP78, GAPDH, and β‐Actin were purchased from Cell Signaling Technology (Boston, MA, USA). Antibody against CIP2A for immunohistochemistry (IHC) was purchased from Novus Biologicals (Littleton, CO, USA), and for western blot analysis was purchased from Santa Cruz Biotechnology (Dallas, TX, USA). Antibody against ERK was purchased from Zymed (Grand Island, NY, USA). Tunicamycin, cycloheximide, 3‐[4, 5‐dimethylthiazol‐ 2‐y1]‐2, 5‐diphenyltetrazolium bromide) (MTT), and 4‐(2‐Aminoethyl) benzenesulfonyl fluoride hydrochloride (AEBSF) were purchased from Sigma‐Aldrich (St. Louis, MO, USA).

### 2.3 Plasmids and constructions

pCGN‐vector and pCGN‐ATF6 (1–373) were purchased from Addgene (Cambridge MA, USA). pCMV‐vector and pCMV‐KIAA1524‐DDK were purchased from Origene (Rockville, MD, USA). The upstream region of the CIP2A promoter from the transcription start site (TSS) was cloned by PCR amplification from 293T cell genomic DNA and then inserted into the pGL3‐basic with BglII and HindIII digestions (forward primer: 5ʹ‐ATC GGC TAG CTG CTG ACA CAT CAT CAC AGA TG‐3ʹ; reverse primer: 5ʹ‐ATC GAG ATC TTC TCA GAA GCT TCC GGA TCC‐3ʹ). The mutagenesis for CIP2A promoter was performed using QuickChange site directed mutagenesis kit (Stratagene, La Jolla, CA, USA) with according to the manufacturer's instructions.

### 2.4 RNA isolation and RT‐PCR

Total RNA was extracted using Trizol reagent (Invitrogen, Carlsbad, CA, USA) according to the manufacturer's instructions and reverse transcribed into cDNA. The gene expression levels were analyzed using RT‐PCR. The primer sequences for CIP2A were 5ʹ‐TGG CAA GAT TGA CCT GGG ATT TGG A‐3ʹ (forward) and 5ʹ‐AGG AGT AAT CAA ACG TGG GTC CTG A‐3ʹ (reverse). The primer sequences for GRP78 were 5ʹ‐CGG GAT CCA TGA AGC TCT CCC TGG TG‐3ʹ (forward) and 5ʹ‐CCC AAG CTT GGG CAA CTC ATC TTT TTC TG‐3ʹ (reverse). The primer sequences for GAPDH were 5′‐TGA AGG TCG GAG TCA ACG GAT TTG GT‐3′ (forward) and 5′‐CAT GTG GGC CAT GAG GTC CAC CAC‐3′ (reverse).

### 2.5 Knockdown of CIP2A

The small hairpin RNA (shRNA) plasmids for luciferase control gene (shLuci) and the CIP2A gene (shCIP2A) were obtained from the RNAi Core Facility at Academia Sinica, Taipei, Taiwan. The shLuci and shCIP2A constructs were made using the pLKO plasmid (http://www.addgene.org/plko), and the target sequences are as follows: 5′‐CAA ATC ACA GAA TCG TCG TAT‐3′ and 5′‐CCA CAG TTT AAG TGG TGG AAA‐3′, respectively.

### 2.6 Western blot analysis

Whole‐cell extracts were prepared using radio immunoprecipitation assay buffer (150 mm NaCl, 50 mm Tris/HCl, 0.1% SDS, 0.5% sodium deoxycholate, 0.1% Triton X‐100) plus protease inhibitor cocktail (Thermo Scientific, Waltham, MA, USA). The protein concentrations were determined using the Bradford assay (Sigma‐Aldrich), and then, samples were diluted in 5 ×  Laemmli buffer [300 mm Tris/HCl pH 6.8, 10% SDS (w/v), 5%, 2‐mercaptoethanol, 25% glycerol (v/v), 0.1% bromophenol blue w/v] and boiled for 5 min. Forty‐five microgram of protein was separated by 8–15% SDS/PAGE and transferred onto polyvinylidene fluoride (PVDF) membranes (PALL Life Science, Ann Arbor, MI, USA). Unspecific binding sites on the PVDF membranes were blocked with 5% nonfat milk in TBST (20 mm Tris/HCl, pH 7.6, 137 mm NaCl, 1% Tween‐20). Membranes were then hybridized with primary antibodies for overnight at 4 °C, followed by incubation with horseradish peroxidase (HRP)‐conjugated secondary antibodies. The membranes were then developed using Immobilon Western chemiluminescence HRP substrates (Millipore, Burlington, MA, USA). Images were capture by Luminescence/Fluorescence Imaging System (GE Healthcare, Pittsburgh, PA, USA).

### 2.7 Cell viability analysis

Cell viability was analyzed using MTT assay. Cells (1 × 10^4^) were seeded on 96‐well culture plates and treated with each experimental condition. At the time point, MTT (0.5 mg·mL^−1^) was added to each well and cells were incubated for 1 h. The blue MTT formazan precipitate was subsequently dissolved in 100 μL of DMSO. The absorbance at 570/650 nm was measured with an ELISA reader (Infinite 200, GMI, Ramsey, MN, USA).

### 2.8 ChIP assay

A ChIP assay was performed as previously described (Hsu *et al*., 2008) using protein A/G agarose and anti‐ATF6 antibody. The immunoprecipitated DNA was used to amplify DNA fragments via PCR with specific primers. The primer sequences for GRP78 were 5′‐GCG GAG CAG TGA CGT TTA TT‐3′ (forward) and 5′‐GAC CTC ACC GTC GCC TAC T‐3′ (reverse). The primer sequences for CIP2A were 5′‐GTC ACA TCG TCA AAG GTG G‐3′ (forward) and 5′‐GAA ACT GCC AGT CAG GGA AC‐3′ (reverse).

### 2.9 CIP2A promoter luciferase activity assay

Transfection and luciferase activity assay were performed as previously described with slight modifications (Hsu *et al*., 2013). Colon cancer cells were plated in 6‐well culture plates and were respectively transfected with pGL3‐basic, pGL3‐Cip2a, and a β‐gal plasmid using lipofectamine 2000 (Invitrogen) according to the manufacturer's instructions. For luciferase activity assays, the cells were transfected with each vector for 18 h and harvested to determine luciferase activity. The assay was carried out using a kit provided by Promega Corp. (Madison, MI, USA) following the manufacturer's instructions. A plasmid encoding β‐galactosidase was used to normalize for transfection efficiency, and relative luciferase activity (defined as reporter activity) was calculated as the ratio of luciferase activity/β‐galactosidase activity.

### 2.10 Patient specimens

All pathological samples from the patients in this study were collected after written informed consent had been obtained. This study was approved by the ethics committee of the Institutional Review Board of Taipei Veterans General Hospital and was conducted in compliance with the Declaration of Helsinki. The experiments were performed in accordance with approved guidelines and regulations. Patients with colon cancer, based on the World Health Organization criteria (WHO) criteria, were enrolled from January 2000 to January 2014 and were then classified according to the American Joint Committee on Cancer (AJCC) staging system (Version 6). Clinical data were obtained from the cancer registry. The OS was defined as the time from the primary resection to death from cancer. The follow‐up period was considered to end in January 2018 or at the time of death of the patient. For defining location of the primary colon tumors, right colon cancer included primary tumor located in the cecum, ascending colon, hepatic flexure or transverse colon, and left colon cancer included those within the splenic flexure, descending colon, sigmoid colon, or rectosigmoid junction (Brule *et al*., 2015).

### 2.11 Immunohistochemistry

For tissue microarray and IHC, the procedures followed manufacturer's instructions. Omission of the primary antibody served as a negative control, and the positive‐immunostaining was evaluated by clinical physician. The intensity of staining was scored as 0, 1, 2, and 3, respectively. The percentage of stained cells was also counted, and final *H*‐score was obtained by multiplying the staining intensity (0–3) by the percentage of positive cells (%). Thus, the *H*‐scores produced a continuous variable that range from 0 to 300 point, and a cutoff point of 150 was used to distinguish between strong (more than or equal to 150) and weak expression (< 150).

### 2.12 Statistical and survival analysis

Data are expressed as mean ± SD or SE. Statistical comparisons were based on nonparametric tests, and statistical significance was defined at *P* < 0.05. The correlations between clinicopathological variables and immunopositivity were analyzed using the χ^2^ test or Fisher's exact test. Survival was estimated using the Kaplan–Meier method. A two‐sided *P* value of < 0.05 was regarded as statistically significant. spss software (version 16.00; SPSS, Chicago, IL, USA) was used for all the statistical analyses.

## 3 Results

### 3.1 CIP2A expression positively correlates with ATF6 expression in both clinical specimens and with ER stress *in vitro*

**Figure 1:**
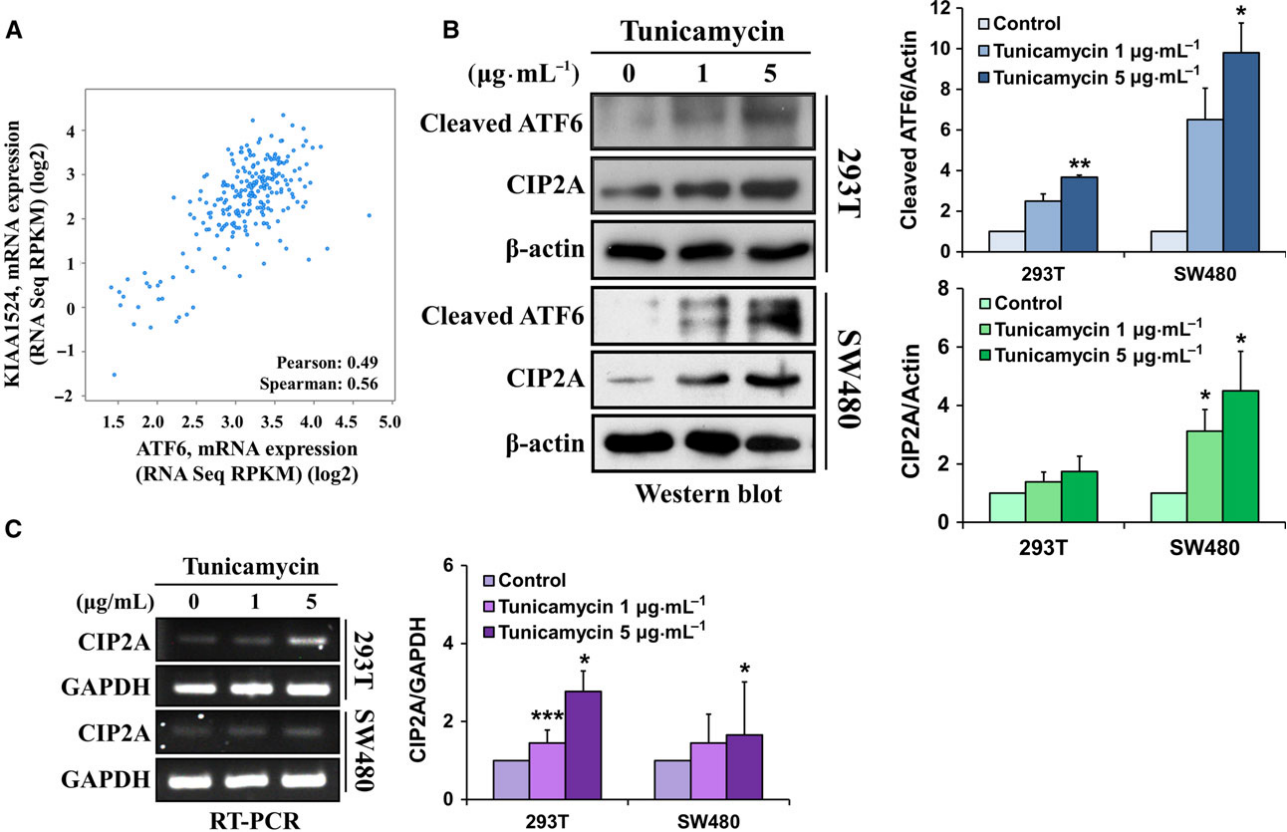
CIP2A expression positively correlates with ATF6 expression in clinical specimens and with ER stress *in vitro*. The mRNA expression level (RPKM, reads per kilobase of exon model per million) of KIAA1524 and ATF6 was downloaded from the cBioPortal website (http://www.cbioportal.org/). The correlation between KIAA1524 and ATF6 was analyzed using Pearson and Spearman's correlation tests, respectively (A). HEK293T and SW480 cells were treated with tunicamycin (0, 1, and 5 μg·mL ^−1^) for 6 h, and the protein (B) and mRNA expressions (C) of ATF6 and CIP2A were analyzed using western blot analysis and RT‐PCR, respectively. Their intensities were quantified and normalized to the internal control β‐actin or GAPDH. Means ± SEM of three independent experiments performed at least in triplicate are shown. Statistical analysis was carried out using Student's *t*‐test. *P < 0.05, **P < 0.01, ***P < 0.001.

To examine the relationship between ATF6 and CIP2A, we downloaded The Cancer Genome Atlas (TCGA) data from the cBioPortal website (http://www.cbioportal.org/) (Cerami *et al*., 2012; Gao *et al*., 2013) and analyzed the correlation between ATF6 and CIP2A gene expression. The results showed that the expression of ATF6 mRNA was positively proportional to the expression of CIP2A mRNA in patients with CRC (Fig. 1A). ER stress induces proteolysis and activation of ATF6. Therefore, we next treated cells with an ER stress inducer, tunicamycin, in both a colon cancer cell line (SW480) and an immortalized normal cell line (HEK293T). We found not only cleaved ATF6 protein expression, but CIP2A protein expression was also increased during ER stress (Fig. 1B). Because cleaved ATF6 serves as a transcriptional factor and regulates downstream gene expression, we next examined the gene expression of CIP2A under tunicamycin treatment. The results showed that CIP2A gene expression was elevated with tunicamycin treatment (Fig. 1C). These results suggest that CIP2A expression positively correlates with ATF6 expression in both clinical specimens and with ER stress *in vitro*.

### 3.2 Active ATF6 induces CIP2A expression without affecting CIP2A protein stability

**Figure 2:**
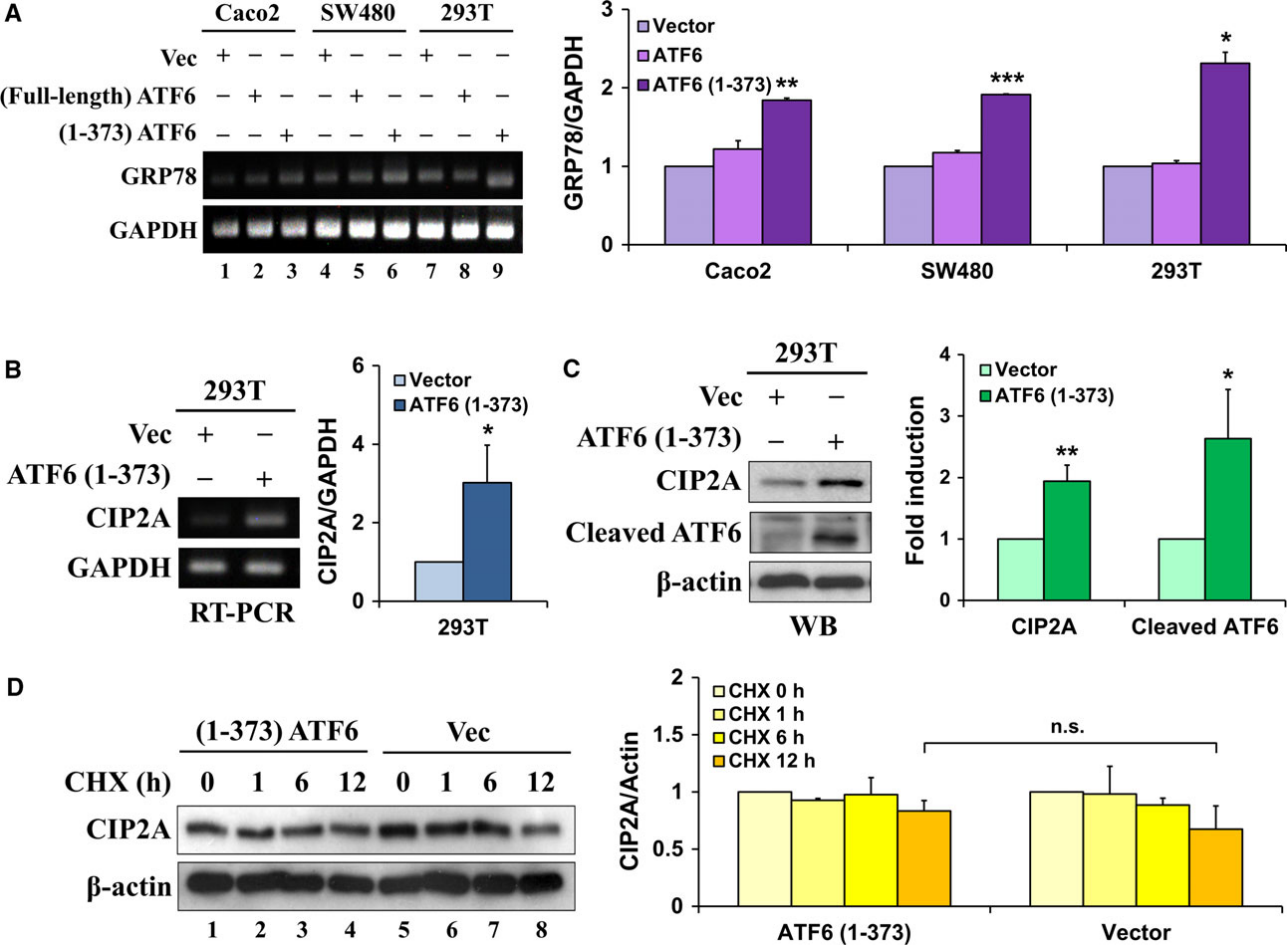
Active ATF6 induces CIP2A expression without affecting CIP2A protein stability. Caco2, SW480, and HEK293T cells were, respectively, transfected with pCGN‐vector, pATF6 (full‐length), and pATF6 (1–373) for 48 h; mRNA expression of GRP78 and GAPDH was analyzed using RT‐PCR (A). HEK293T cells were transfected with either pCGN‐vector or pATF6 (1–373) for 48 h, and mRNA (B) and protein (C) expressions of CIP2A and GAPDH were analyzed using RT‐PCR and western blot, respectively. After transfecting either pCGN‐vector or pATF6 (1–373) for 48 h, HEK293T cells were incubated with 100 μm cycloheximide (CHX, translational inhibitor) for 0, 1, 6, and 12 h. Whole‐cell extracts were subjected to western blot analysis using antibodies specific for CIP2A and β‐actin (D). Their intensities were quantified and normalized to the internal control β‐actin or GAPDH. Means ± SEM of three independent experiments performed at least in triplicate are shown. Statistical analysis was carried out using Student's *t*‐test. *P < 0.05, **P < 0.01, ***P < 0.001.

To explore the possible mechanism by which ATF6 regulates CIP2A gene expression, we transfected colon cancer cell lines (Caco2 and SW480) and HEK293T cells with full‐length ATF6 and active‐form ATF6 (1–373), respectively. The results showed that the mRNA expression of GRP78, which is the targeted gene of ATF6, was increased after expressing full‐length and active ATF6 (1–373) in colon cancer or HEK293T cell lines (Fig. 2A; lanes 3, 6, and 9) (Haze *et al*., 1999). In addition, we found that exogenous expression of ATF6 (1–373) remarkably induced CIP2A mRNA expression (Fig. 2B), as well as protein expression (Fig. 2C). To further evaluate the involvement of the proteasomal degradation of CIP2A in cells during the expression of ATF6 (1–373), HEK293T cells were transfected with ATF6 (1–373) before incubation with cycloheximide (CHX, a protein translational inhibitor). The protein stability of CIP2A was not significantly changed in the presence of exogenous ATF6 (1–373) (Fig. 2D). These results indicated that active ATF6 induced CIP2A expression without affecting its protein stability.

### 3.3 ATF6 directly regulates CIP2A gene transcription through binding to the CIP2A promoter

**Figure 3:**
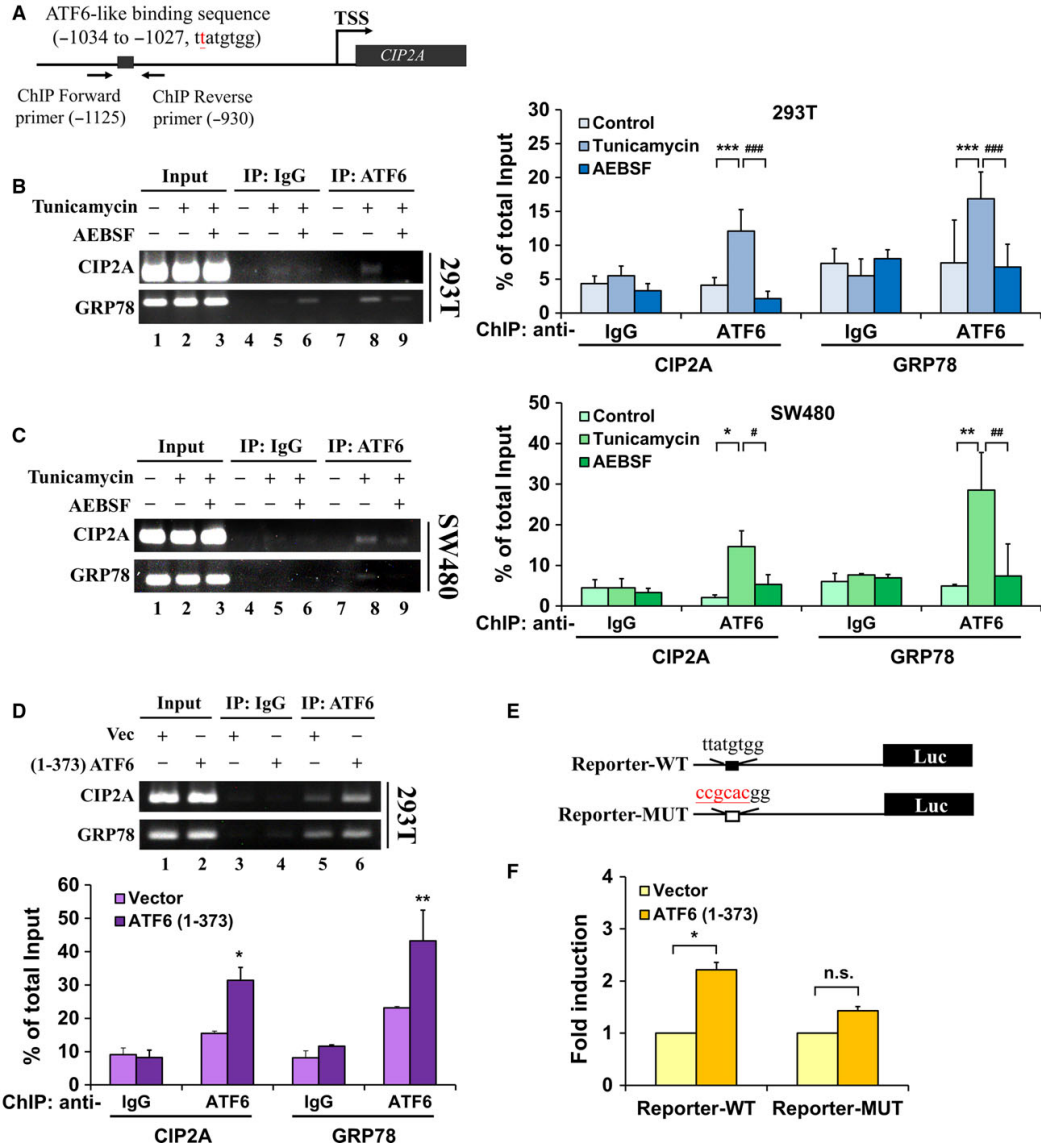
ATF6 directly regulates CIP2A gene transcription through binding to the CIP2A promoter. Scheme representing the region for ChIP primers flanking within the CIP2A promoter (A). HEK293T (B) and SW480 cells (C) were treated with tunicamycin (5 μg·mL^−1^) or AEBSF for 6 h; cells were harvested for ChIP assays using anti‐IgG and anti‐ATF6 antibodies, and precipitated DNA was used to amplify the PCR product of CIP2A and GRP78 promoters. After transfecting either pCGN‐vector or pATF6 (1–373) in HEK293T cells for 48 h, the DNA‐binding ability of ATF6 to the CIP2A and GRP78 promoters was analyzed using ChIP assays (D). The quantitative and statistical analysis was shown. Means of three independent experiments performed at least in triplicate are shown. Scheme representing the constructs of the CIP2A reporter (E). After transfection with either pCGN‐vector or pATF6 (1–373) for 48 h, cells were harvested, and CIP2A‐dependent reporter gene activity was measured by luciferase assay (F). The means ± SEM of three independent experiments performed in triplicate are shown. Statistical analysis was carried out using Student's *t*‐test. *P < 0.05, **P < 0.01, ***P <* *0.001; ^#^ *P *< 0.05, ^##^ *P *< 0.01, ^###^ *P *< 0.001.

Interestingly, we found a UPR element (UPRE)‐like sequence (TTATGTGG in contrast to the canonical UPRE TGACGTGG/A), which is known to be an ATF6‐binding sequence (Wang *et al*., 2000) within the CIP2A promoter (Fig. 3A). To examine whether ATF6 controls CIP2A gene expression through direct binding to the CIP2A promoter, we treated both HEK293T and SW480 cells with tunicamycin, and the DNA‐binding ability of ATF6 to the CIP2A promoter was examined using a ChIP assay (Fig. 3A). The results revealed that ATF6 bound to the CIP2A as well as the GRP78 gene promoters in both HEK293T (Fig. 3B; lane 8) and SW480 cells (Fig. 3C; lane 8) during ER stress, and the binding could be inhibited by AEBSF, which is a blocker for the process of ATF6 proteolysis (Fig. 3B,C; lane 9) (Ye *et al*., 2000). In addition, the DNA‐binding ability of ATF6 to the CIP2A gene promoter was increased after the exogenous expression of active ATF6 in HEK293T cells (Fig. 3D). To investigate whether the putative ATF6 targeting sequence (TTATGTGG) is crucial for the binding of ATF6 to the CIP2A gene promoter, the putative (TTATGTGG) and mutant (CCGCACGG) sequences within the CIP2A gene promoter were, respectively, cloned into a luciferase reporter vector (Fig. 3E) and transiently co‐transfected with active ATF6 into HEK293T cells. Co‐transfection with active ATF6 and the ‘TTATGTGG’ vector resulted in increased promoter activity compared with the control vector, and the promoter activity was attenuated when ‘TTATGTGG’ was replaced by the mutant ‘CCGCACGG’ (Fig. 3F). These results suggested that ATF6 directly regulated CIP2A gene transcription through binding to the CIP2A promoter.

### 3.4 CIP2A sustains the survival of colon cancer cells through ATF6 under both basal and ER stress conditions

**Figure 4:**
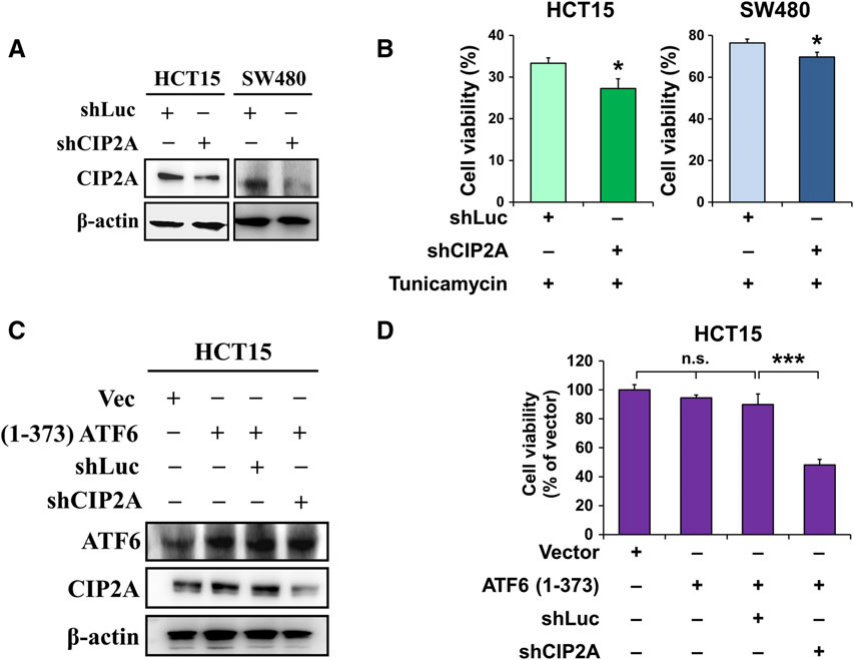
CIP2A sustains survival of colon cancer cells through ATF6 under both of basal and ER stress conditions. HCT15 and SW480 cells were transfected with shRNA against luciferase or CIP2A for 48 h, and whole‐cell extracts were subjected to western blot analysis using antibodies specific for CIP2A and β‐actin (A) and to MTT assays (B). HCT15 cells were co‐transfected with ATF6 (1–373)‐expressing or control plasmid and shRNA against CIP2A (shCIP2A) or luciferase (shLuc) for 48 h. The transfected cells were further analyzed by western blotting using anti‐ATF6, anti‐CIP2A, and anti‐β‐actin antibodies (C) and by MTT assays (D). The means ± SEM of three independent experiments performed in triplicate are shown. Statistical analysis was carried out using Student's *t*‐test. *P < 0.05, ***P < 0.001.

Due to the importance of ATF6 signaling in the survival of cancer cells, we next evaluated the effect of CIP2A knockdown in colon cancer cells. The results showed that knockdown of CIP2A reduced cell viability in the presence of tunicamycin in HCT15 and SW480 cells (Fig. 4A,B), indicating that basal expression of CIP2A was necessary for colon cancer survival in ER stress. Overexpression of the active form of ATF6 increased CIP2A expression, which was repressed by CIP2A shRNA transfection (Fig. 4C). In addition, knockdown of CIP2A suppressed cell viability in active ATF6‐expressing HCT15 cells (Fig. 4D). A previous study indicated that knockdown of CIP2A increased the sensitivity to chemotherapy such as 5‐FU (Teng *et al*., 2012). Our data also showed that pretreatment with tunicamycin resisted 5‐FU‐suppressed cell viability (Fig. S1). The results indicated that CIP2A sustains the survival of colon cancer cells through ATF6 under ER stress conditions.

### 3.5 Clinical significance of ATF6 and CIP2A expression in colon cancer

**Figure 5:**
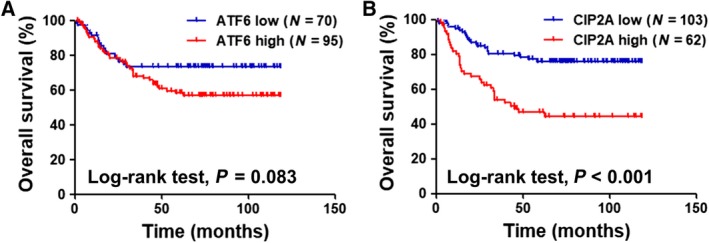
Clinical significance of ATF6 and CIP2A expression in colon cancer. OS rates of patients with colon cancer are plotted against time (months) for different parameters: (A) low and high expression of ATF6 protein (*P *= 0.083) and (B) low and high expression of CIP2A protein (*P *< 0.001).

To determine the clinical significance of ATF6 and CIP2A expression in colon cancer, we analyzed the relationship between ATF6 protein expression and various clinicopathological parameters in a total of 165 patients with colon cancer. We further included nine patients with rectal cancer (total *N* = 174; Tables S1 and S2), for exploratory analysis. ATF6 protein expression was positively correlated with CIP2A protein expression (*P *< 0.001) and tumor stages (*P *= 0.007). However, ATF6 protein expression was not correlated with other clinicopathological parameters such as age, gender, tumor location, pathology, tumor grade, or lymphovascular involvement in patients with colon cancer (Table 1) and CRC (Table S1). The clinicopathological characteristics in multivariate analysis by Cox regression, ATF6, were not a prognostic factor for survival, while CIP2A was independently associated with survival in patients with colon cancer (Table 2) and CRC (Table S2). We further investigated the prognostic value of ATF6 and CIP2A expression, independently, in patients with colon cancer and CRC. The results showed that the expression of ATF6 level did not associated with OS (*P *= 0.083; Fig. 5A), while colon cancer patients with strong expression of CIP2A showed significantly lower OS rates than those with weak expression (*P *< 0.001; Fig. 5B). CRC patients with strong expression of CIP2A also exhibited significantly lower OS rates than those with weak expression (Fig. S2). Collectively, these results indicated that CIP2A may be a prognosis marker in the clinical prognosis of patients with colon cancer.

**Table 1:** The relationships between ATF6 expression and clinical variables in patients with colon cancer (*N* = 165)

	ATF6 weak expression	ATF6 strong expression	*P* value
	*n* (%)	*n* (%)	
Age (year)			
≤ 65	31 (44.3)	42 (44.2)	0.992
> 65	39 (55.7)	53 (55.8)	
Gender			
Female	21 (30.0)	36 (37.9)	0.292
Male	49 (70.0)	59 (62.1)	
Location			
Left colon	40 (57.1)	53 (55.8)	0.862
Right colon	30 (42.9)	42 (44.2)	
Stage AJCC VI			
I	11 (15.7)	6 (6.3)	0.007^a^
II	30 (42.9)	26 (27.4)	
III	13 (18.6)	37 (38.9)	
IV	16 (22.9)	26 (27.4)	
Pathology			
Adenocarcinoma	69 (98.6)	94 (98.9)	0.827
Mucinous adenocarcinoma	1 (1.4)	1 (1.1)	
Grade			
Low	64 (91.4)	84 (88.4)	0.530
High	6 (8.6)	11 (11.6)	
Lymphovascular involvement			
No	59 (84.3)	73 (76.8)	0.237
Yes	11 (15.7)	22 (23.2)	
CIP2A expression			
Weak	56 (80.0)	47 (49.5)	< 0.001^a^
Strong	14 (20.0)	48 (50.5)	

a *P *< 0.05.

**Table 2:** Prognostic factors for survival in patients with colon cancer according to univariate and multivariate analyses in the Cox proportional hazards model (*N* = 165)

Variable	Univariate analysis			Multivariate analysis		
	Hazard ratio	95% CI	*P* value	Hazard ratio	95% CI	*P* value
Age > 65 y/o	0.844	0.621–1.791	1.054	0.903	0.509–1.602	0.728
Gender	1.004	0.577–1.746	0.988	1.187	0.651–2.165	0.576
Stage AJCC VI	4.494	3.594–8.398	< 0.001^a^	5.629	3.515–9.013	< 0.001^a^
Sideness	0.815	0.627–1.060	0.127	0.785	0.590–1.045	0.097
Lymphovascular invasion	3.619	2.113–6.196	< 0.001^a^	1.897	1.071–3.360	0.028^a^
High grade	1.454	0.688–3.076	0.327	0.977	0.427–2.231	0.955
ATF6	1.632	0.931–2.860	0.087	0.688	0.363–1.305	0.253
CIP2A	2.948	1.729–5.029	< 0.001^a^	3.110	1.643–5.886	< 0.001^a^

a *P *< 0.05.

## 4 Discussion

In the present study, we demonstrated that the ATF6‐CIP2A pathway is essential to colon cancer cell survival, and CIP2A serves as a biomarker in clinical prognosis. We found that the expression of ATF6 was positively correlated with the expression of CIP2A not only in patients with colon cancer but also in both the colon cancer cell line and the immortalized normal cell line under ER stress. Our study revealed that ATF6 could directly bind to the CIP2A promoter and regulate CIP2A gene expression. In addition, the expression of CIP2A was crucial to sustain colon cancer cell survival under ER stress. Moreover, CIP2A protein expression determined the OS rates in patients with colon cancer. Taken together, CIP2A is a promising biomarker for the prognosis of patients with colon cancer, and targeting ATF6‐CIP2A signaling might be a strategy for colon cancer therapy.

Cumulative evidence has indicated that ER stress, especially GRP78, which is a downstream target of ATF6, was correlated with chemotherapy resistance in cancer cells (Garg *et al*., 2015; Kim *et al*., 2016; Wang and Kaufman, 2014). In our study, we found that tunicamycin treatment and exogenous expression of active ATF6 (1–373) induced both the mRNA and protein expression of CIP2A in colon cancer cells (Figs 1 and 2). In addition, we also found that knockdown of CIP2A enhanced the tunicamycin‐induced cytotoxicity in colon cancer cells (Fig. 4). The results determined that tunicamycin treatment contributed to 5‐FU resistance (Fig. S1). Further investigation is warranted to determine whether knockdown of CIP2A could sensitize colon cancer cells to chemotherapy.

Activating transcription factor 6 is an ER stress sensor located in the ER membrane. Upon stimulation of ER stress, ATF6 translocates from the ER to the Golgi membrane, in which ATF6 is cleaved by site‐1 protease (S1P) and site‐2 protease (S2P) to release the active form of ATF6 (N terminus) that functions as a transcriptional factor (Ye *et al*., 2000). ATF6 is known to regulate ER stress response genes by recognizing a specific DNA sequence called the ER stress response element (ERSE, CCAAT‐N_9_‐CCACG) (Haze *et al*., 1999; Yoshida *et al*., 1998) and the unfolded protein response element [UPRE, (G)(G)TGACGTG(G/A)], where the nucleotides in parentheses are less strongly maintained (Wang *et al*., 2000). A recent study used bioinformatics analysis to reveal that ATF6 could also bind to the UPRE‐like sequence ‘TATGTGG’ within the insulin II promoter and regulate its gene expression (Amyot *et al*., 2011). In the present study, we found a novel sequence ‘TTATGTGG’ within the CIP2A promoter responsible for ATF6 binding (Fig. 3). Our results provided a new parameter in search of unknown targets for ATF6.

Cancerous inhibitor of protein phosphatase 2A was upregulated and was negatively proportional to clinically prognostic features in various types of cancer, including CRC (Bockelman *et al*., 2011; Dong *et al*., 2011; Liu *et al*., 2014; Teng *et al*., 2012). Previous studies demonstrated that knockdown of CIP2A reduced c‐Myc expression and cell growth of CRC cells. The level of CIP2A mRNA was significantly correlated with advanced tumor stages and OS (Wiegering *et al*., 2013). In contrast to the findings, CIP2A had been reported that is not an independent prognostic factor in CRC but was associated nuclear c‐Myc (Bockelman *et al*., 2012). In addition, high level of CIP2A in CRC patients with wild‐type KRAS was correlated with short OS after colorectal liver metastasectomy (Chen *et al*., 2015). CIP2A expression contributes to radiotherapy resistance in rectal cancer (Birkman *et al*., 2018). In the present study, we found that in patients with colon cancer, either ATF6 or CIP2A protein overexpression were negatively correlated to the OS rates (Fig. 5). In addition, we also found that CIP2A rather than ATF6 was the important factor to predict the OS rates in the clinic. The results confirmed that CIP2A was crucial to CRC patient survival in our previous study (Teng *et al*., 2012). Moreover, other transcriptional factors, such as Elk1 and Ets1, have been reported to control CIP2A gene expression in cervical cancer, endometrial carcinoma, and triple‐negative breast cancer (Liu *et al*., 2017; Pallai *et al*., 2012). This is the first study that provides the link between ATF6 and CIP2A in cancer. We further included nine patients with rectal cancer (total *N* = 174; Tables S1 and S2), and the analysis was similar with colon cancer patients only (*N* = 165). However, the limited number of patients with rectal cancer makes us difficult to draw a conclusion on the population of rectal cancers, and therefore, our study is limited to the population of patients with colon cancer, whether the significance of CIP2A and ATF6 applied to patients with rectal cancer need further studies. Moreover, further investigation is required to determine whether ATF6‐CIP2A signaling also plays an important role in other types of cancer.

## 5 Conclusion

In conclusion, our present study provides evidence of linkage between ER stress and CIP2A, while conditional ER stress‐related ATF6 upregulates CIP2A and contributes to the prognosis of colon cancer. These findings suggest that targeting CIP2A may be implicated in disrupting ER stress in mediating colon cancer cell survival and thus poor prognosis.

## Author contributions

H‐WT and C‐YL were responsible for coordination and manuscript editing. C‐CH, T‐TH, and JLC drafted the manuscript. C‐CH, T‐TH, C‐HL, J‐LC, S‐HY, J‐KJ, W‐SC, and K‐DL conducted *in vitro* experiments. H‐WT conducted histopathological experiments. C‐YL, J‐LC, and H‐WT performed or helped clinical data acquisition and analysis. C‐YL, C‐CH, T‐TH, C‐HL, J‐LC, and H‐WT helped in data interpretation and statistical analysis. All authors had substantial contributions to the conception or design of the work. All authors read the final manuscript. All authors agreed with the accuracy and integrity of all parts of the work.

## Supporting information


**Fig. S1.** Tunicamycin treatment restored 5‐FU‐reduced cell viability of SW480 cells.
**Fig. S2.** Clinical significance of CIP2A and ATF6 expression in patients with colorectal cancer.
**Table S1.** The relationships between ATF6 expression and clinical variables in patients with colorectal cancer.
**Table S2.** Prognostic factors for survival in patients with colorectal cancer according to univariate and multivariate Analyses in the Cox proportional hazards model.
