# Supplementary material for: ER stress‐related ATF6 upregulates CIP2A and contributes to poor prognosis of colon cancer
Source: Mol Oncol. 2018 Aug 20;12(10):1706–17. doi: 10.1002/1878-0261.12365 (PMC6166000; doi:10.1002/1878-0261.12365)
Supplement: Supplementary file 1 — Fig. S1. Tunicamycin treatment restored 5‐FU‐reduced cell viability of SW480 cells. Fig. S2. Clinical significance of CIP2A and ATF6 expression in patients with colorectal cancer. Table S1. The relationships between ATF6 expression and clinical variables in patients with colorectal cancer. Table S2. Prognostic factors for survival in patients with colorectal cancer according to univariate and multivariate Analyses in the Cox proportional hazards model. [file MOL2-12-1706-s001.docx]

**Title:**

**ER stress related ATF6 upregulates CIP2A and contributes to poor prognosis of colon cancer**

Chun-Yu Liu^1,2,3^, Chia-Chi Hsu^1,3^, Tzu-Ting Huang^1,3^, Chia-Han Lee^1^, Ji-Lin Chen^1,3^, [Shung-Haur Yang](http://link.springer.com/search?facet-author=%22Shung-Haur+Yang%22) ^2, 4^, [Jeng-Kai Jiang](http://link.springer.com/search?facet-author=%22Jeng-Kai+Jiang%22)^2, 4^, [Wei-Shone Chen](http://link.springer.com/search?facet-author=%22Wei-Shone+Chen%22)^2, 4^, Kuan-Der Lee^5,6^, Hao-Wei Teng^1,2*^

^1^Division of Medical Oncology, Center for Immuno-Oncology, Department of Oncology, Taipei Veterans General Hospital, No. 201, Sec. 2, Shih-Pai Road, Taipei 112, Taiwan

^2^School of Medicine, National Yang-Ming University, No. 155, Sec. 2, Li-Nong Street, Taipei 112, Taiwan

^3^Comprehensive Breast Health Center, Taipei Veterans General Hospital, No. 201, Sec. 2, Shih-Pai Road, Taipei 112, Taiwan

^4^Division of Colon and Rectum Surgery, Department of Surgery, Taipei Veterans General Hospital, Taipei, No. 201, Sec. 2, Shih-Pai Road, Taipei 112, Taiwan ^5^Division of Hematology and Oncology, Department of Internal Medicine, Taipei Medical University Hospital.

^6^School of Medicine, Taipei Medical University.

***Correspondence Author:**

Hao-Wei Teng, MD, PhD

Division of Medical Oncology, Department of Oncology, Taipei Veterans General Hospital, No. 201, Sec. 2, Shih-Pai Road, Taipei 112, Taiwan and School of Medicine, National Yang-Ming University, No. 155, Sec. 2, Li-Nong Street, Taipei 112, Taiwan. Email: hwteng1971@gmail.com TEL: +886-953255179

| **Supporting Information**  **Table S1. The relationships between ATF6 expression and clinical variables in patients with colorectal cancer (N=174).** | | | | | | |
| --- | --- | --- | --- | --- | --- | --- |
|  |  | ATF6 weak expression | | ATF6 strong expression | | *P* value |
|  |  | n | (%) | n | (%) |  |
| Age (year) | <= 65 | 35 | (46.7) | 43 | (43.4) | 0.671 |
|  | >65 | 40 | (53.3) | 56 | (56.6) |  |
| Gender | Female | 22 | (29.3) | 36 | (36.4) | 0.330 |
|  | Male | 53 | (70.7) | 63 | (63.6) |  |
| Location | Left colon | 41 | (54.7) | 53 | (53.5) | 0.695 |
|  | Right colon | 29 | (38.7) | 42 | (42.4) |  |
|  | Rectum | 5 | (6.7) | 4 | (4.0) |  |
| Stage AJCC VI | I | 11 | (14.7) | 6 | (6.1) | 0.008 |
|  | II | 30 | (40.0) | 26 | (26.3) |  |
|  | III | 13 | (17.3) | 37 | (37.4) |  |
|  | IV | 21 | (28.0) | 30 | (30.3) |  |
| Pathology | Adenocarcinoma | 74 | (98.7) | 97 | (98.0) | 0.119 |
|  | Mucinous adenocarcinoma | 1 | (1.3) | 2 | (2.0) |  |
| Grade | Low | 69 | (92.0) | 87 | (87.9) | 0.377 |
|  | High | 6 | (8.0) | 12 | (12.1) |  |
| Lymphvascular involvement | No | 63 | (84.0) | 76 | (76.8) | 0.239 |
|  | Yes | 12 | (16.0) | 23 | (23.2) |  |
| CIP2A expression | Weak | 59 | (78.7) | 47 | (47.5) | <0.001 |
|  | Strong | 16 | (21.3) | 52 | (52.5) |  |
| Abbreviation: CIP2A, Cancerous Inhibitor of Protein phosphatase 2A; AJCC, American Joint Committee on Cancer. | | | | | | |

| **Table S2. Prognostic factors for survival in patients with colorectal cancer according to univariate and multivariate Analyses in the Cox proportional hazards model (N=174).** | | | | | | | |  |  |
| --- | --- | --- | --- | --- | --- | --- | --- | --- | --- |
| Variable | | Univariate Analysis | | | Multivariate Analysis | | | | |
|  | | Hazard ratio | 95% CI | *P* value | | Hazard ratio | 95% CI | *P* value | |
| Age >65y/o | | 0.962 | 0.583-1.587 | 0.880 | | 0.901 | (0.523-1.551) | 0.707 | |
| Gender | | 1.124 | (0.655-1.929) | 0.671 | | 1.196 | (0.665-2.148) | 0.550 | |
| Stage AJCC VI | | 5.554 | (3.662-8.423) | <0.001* | | 5.679 | (3.608-8.938) | <0.001* | |
| Sideness | | 0.876 | (0.682-1.124) | 0.296 | | 0.773 | (0.590-1.014) | 0.063 | |
| LVSI | | 3.348 | (2.004-5.592) | <0.001* | | 1.723 | (0.992-2.993) | 0.054 | |
| Grade | | 1.472 | 0.726-2.985 | 0.284 | | 1.032 | (0.477-2.232) | 0.936 | |
| ATF6 | | 1.529 | (0.904-2.588) | 0.107 | | 0.635 | (0.343-1.174) | 0.147 | |
| CIP2A | | 2.850 | (1.713-4.741) | <0.001* | | 2.985 | (1.611-5.332) | 0.001* | |
| Abbreviation: LVSI, lymphovascular invasion; CIP2A, Cancerous Inhibitor of Protein phosphatase 2A; AJCC, American Joint Committee on Cancer, ATF6, activating transcription factor 6  **P* < 0.05 | | | | | | | | | |

**
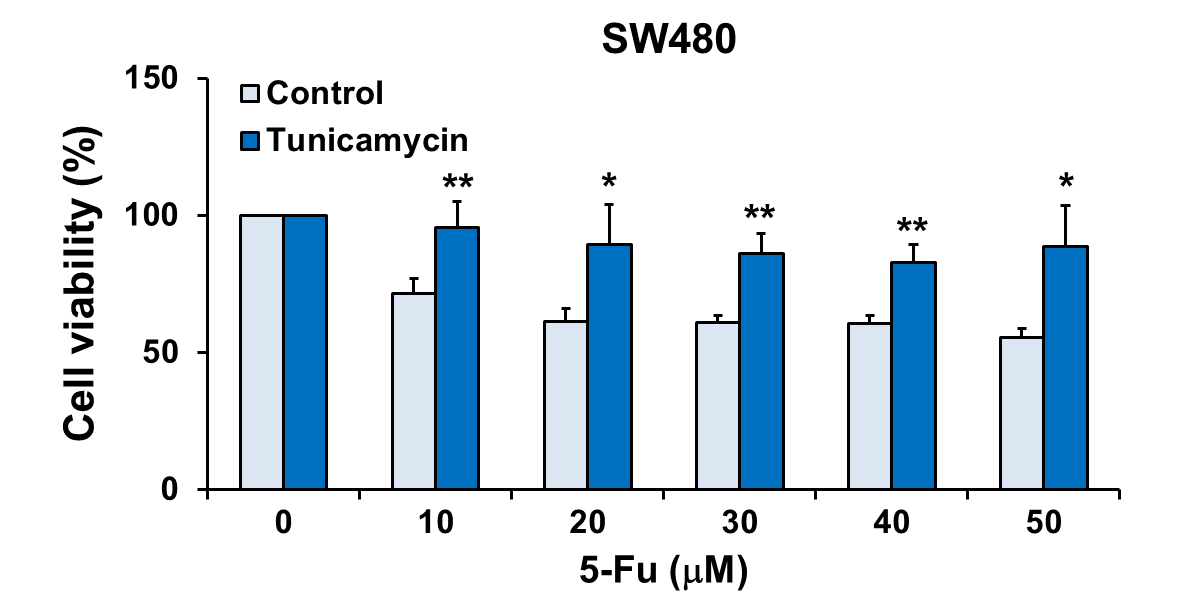
**

**Figure S1. Tunicamycin treatment restored 5-FU-reduced cell viability of SW480 cells.**

SW480 cells were pretreated with tunicamycin (5 μg/ml) or vehicle for 6 h, the treated cells were further treated with 5-FU at indicated concentration for 48 h. The cell viability was measured by MTT assay. The means ± SEM of three independent experiments performed in triplicate are shown. Statistical analysis was done using Student’s *t*-test. **P*<0.05, ***P*<0.01.


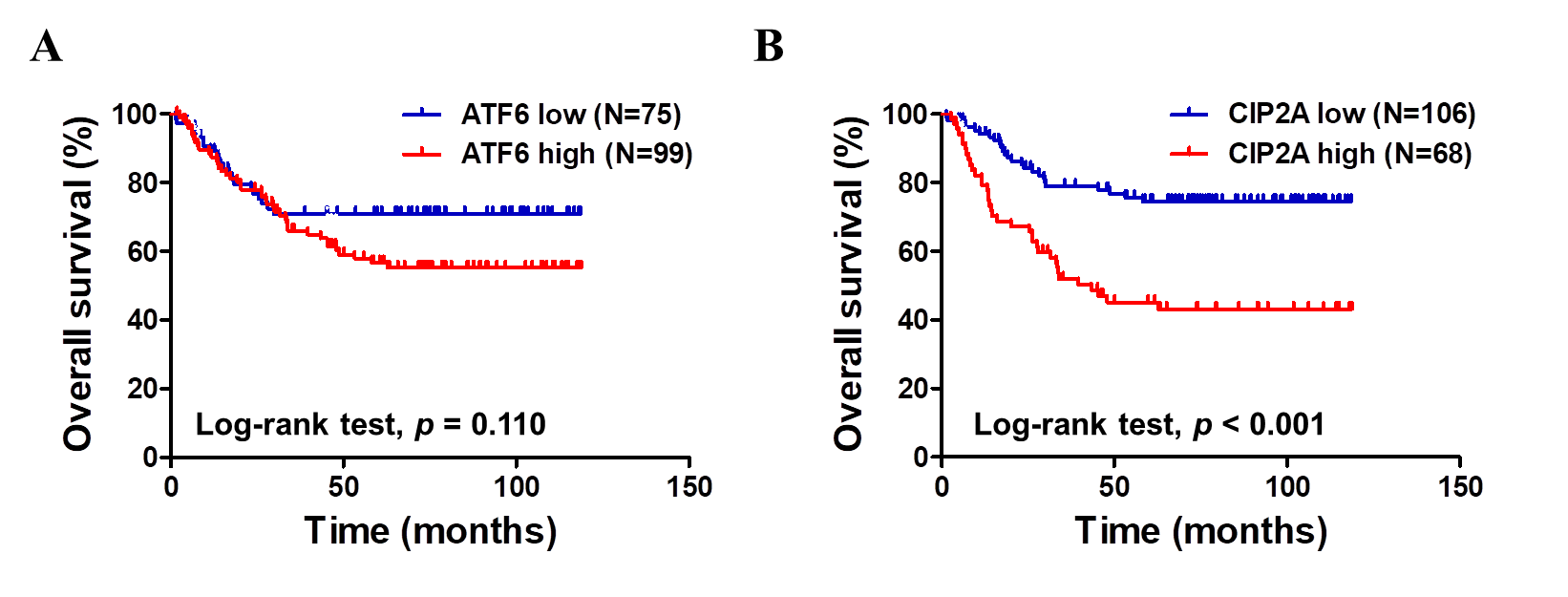


**Figure S2. Clinical significance of CIP2A and ATF6 expression in patients with colorectal cancer.**

Overall survival rates of patients with colorectal cancer are plotted against time (months) for different parameters: **(A)** strong (N=99) and weak (N=75) expression of ATF6 protein (***P*=0.110**) and **(B)** strong (N=68) and weak (N=106) expression of CIP2A protein (***P*<0.001**).
